# PARTICIPATION OUTSIDE HOME FOR PEOPLE LIVING WITH DEMENTIA: DO THEY REALLY EXPERIENCE A SHRINKING WORLD?

**DOI:** 10.1093/geroni/igz038.2848

**Published:** 2019-11-08

**Authors:** Isabel Margot-Cattin, Nicolas Kühne, Catherine Ludwig, Louise Nygard, Anders Kottorp

**Affiliations:** 1 University of Applied Sciences and Arts of Western Switzerland (HES-SO), Haute école de travail social et de la santé (EESP), Lausanne, Vaud, Switzerland; 2 University of Applied Sciences and Arts of Western Switzerland, Lausanne, Vaud, Switzerland; 3 University of Applied Sciences and Arts of Western Switzerland, Geneva, Geneve, Switzerland; 4 Karolinska Institutet, Huddinge, Stockholms Lan, Sweden; 5 Malmö University, Malmö, Skane Lan, Sweden

## Abstract

Accessing places outside home where activities are performed provides both benefits (e.g. participation in activities), and challenges (e.g. finding one’s way) for people with cognitive deficits. Participation in these places appears to depend on various factors such as the living situation of the person, availability of commodities and supporting social networks, or preserved ability to drive. However, clear patterns of participation remain scarcely documented. This study addresses the need for understanding of participation outside home among people with dementia through the places they visit. The aim is to describe how the outside world may be shrinking for them. People with and without dementia (n=70), aged 65+, were interviewed using the Participation in ACTivities and Places OUTside the Home (ACT-OUT) questionnaire across Switzerland. Results show that people with dementia participate significantly less in commercial, social and cultural places, but visit medical care places at a higher level, than those without dementia.

